# Xenotransplantation: Current Status in Preclinical Research

**DOI:** 10.3389/fimmu.2019.03060

**Published:** 2020-01-23

**Authors:** Tianyu Lu, Bochao Yang, Ruolin Wang, Chuan Qin

**Affiliations:** ^1^Institute of Laboratory Animal Sciences, Chinese Academy of Medical Sciences and Comparative Medicine Center, Peking Union Medical College, Beijing, China; ^2^NHC Key Laboratory of Human Disease Comparative Medicine, The Institute of Laboratory Animal Sciences, Chinese Academy of Medical Sciences & Peking Union Medical College, Beijing, China; ^3^Beijing Engineering Research Center for Experimental Animal Models of Human Critical Diseases, Beijing, China

**Keywords:** immunological rejection, coagulation dysfunction, genetically modified pigs, non-human primate, xenotransplantation

## Abstract

The increasing life expectancy of humans has led to a growing numbers of patients with chronic diseases and end-stage organ failure. Transplantation is an effective approach for the treatment of end-stage organ failure; however, the imbalance between organ supply and the demand for human organs is a bottleneck for clinical transplantation. Therefore, xenotransplantation might be a promising alternative approach to bridge the gap between the supply and demand of organs, tissues, and cells; however, immunological barriers are limiting factors in clinical xenotransplantation. Thanks to advances in gene-editing tools and immunosuppressive therapy as well as the prolonged xenograft survival time in pig-to-non-human primate models, clinical xenotransplantation has become more viable. In this review, we focus on the evolution and current status of xenotransplantation research, including our current understanding of the immunological mechanisms involved in xenograft rejection, genetically modified pigs used for xenotransplantation, and progress that has been made in developing pig-to-pig-to-non-human primate models. Three main types of rejection can occur after xenotransplantation, which we discuss in detail: (1) hyperacute xenograft rejection, (2) acute humoral xenograft rejection, and (3) acute cellular rejection. Furthermore, in studies on immunological rejection, genetically modified pigs have been generated to bridge cross-species molecular incompatibilities; in the last decade, most advances made in the field of xenotransplantation have resulted from the production of genetically engineered pigs; accordingly, we summarize the genetically modified pigs that are currently available for xenotransplantation. Next, we summarize the longest survival time of solid organs in preclinical models in recent years, including heart, liver, kidney, and lung xenotransplantation. Overall, we conclude that recent achievements and the accumulation of experience in xenotransplantation mean that the first-in-human clinical trial could be possible in the near future. Furthermore, we hope that xenotransplantation and various approaches will be able to collectively solve the problem of human organ shortage.

## Introduction

Transplantation is an effective approach for the treatment of end-stage organ failure. However, the imbalance between supplement and demand for human organs is a bottleneck for clinical transplantation. According to the US Government Information on Organ Donation and Transplantation, more than 113,000 candidates were on the transplant waiting list as of January 2019, while only 36,528 transplants were performed in 2018 [data available from URL: https://www.organdonor.gov/statistics-stories/statistics.html (accessed June 29, 2019)]. In China, more than 300,000 people are on the waiting list, but only ~16,000 organs are available each year (1). Xenotransplantation may be an alternative solution to this grave problem. The World Health Organization (WHO) defines xenotransplantation as “any procedure that involves the transplantation, implantation or infusion into a human recipient of either: (i) live cells, tissues, or organs from a non-human animal source; or (ii) human body fluids, cells, tissues or organs that have had *ex vivo* contact with live non-human animal cells, tissues or organs” [Xenotransplantation, WHO, Geneva, Switzerland 2016. Available from URL: http://www.who.int/transplantation/xeno/en/ (accessed 2019 June 29)].

Xenotransplantation is not a new concept. It was first mentioned in 1667 in the context of the xenotransfusion of blood from lambs to humans (2). Clinical use of animal organs has also been documented, such as the transplantation of a rabbit kidney to a human in 1905 (3). Because non-human primates (NHPs) are phylogenetically closer to humans than are other species, several trials involving the kidneys, hearts, and livers of NHPs were conducted from the 1920s to 1990s (4, 5). However, researchers found that NHPs were not suitable source animals for clinical xenotransplantation because of ethical concerns, the high risk of cross-species transmission of infections to humans, difficulties in breeding, organ size disparities, and other impracticalities (6). Since the 1990s, researchers have attempted to use pigs as the source animal for xenotransplantation, and the pig is currently considered the most appropriate candidate species. Reasons for selecting the pig as a source animal include the pig's relatively large litter size and short maturation period, its size and physiological similarity to humans, the low risk of xenozoonosis, and the readily application of genetic engineering techniques to produce porcine organs that are resistant to rejection (7). However, the genetic discrepancy between pigs and humans has resulted in barriers for xenotransplantation, including immunological rejection, and risk of xenozoonosis. As with human allotransplants, xenotransplants are prone to immunological rejection. However, a vascularized porcine organ is more vigorously rejected in comparison with the current reaction observed in allotransplants because of the genetic distance between pigs and primates. Thanks to genetically modified pigs and immunosuppressive therapy, survival time results for xenografts have improved considerably in preclinical xenotransplantation models. These results in NHP models indicate that the use of xenotransplantation in clinical applications is approaching.

In this article, we (a) describe our understanding of immunological rejection responses in xenotransplantation, (b) summarize the genetically modified pigs used for xenotransplantation, and (c) report the current survival time of xenografts in pig-to-NHP models. On the basis of this considerable progress, we hold that clinical application of xenotransplantation will soon be a reality.

## Immunological Barriers for Xenotransplantation

Some decellularized extracellular matrix products, such as cornea and cardiac valves, have been used in clinical settings (8, 9). However, these grafts have largely been structural tissues from which the pig cells have been removed. The tissues are repopulated with human recipient cells after transplantation. Vascularized organ and cell transplantation have been impeded by rejection. Immune responses following discordant xenotransplantation include both acquired immunity and innate immunity, in which natural antibodies, complement, natural killer (NK) cells, and macrophages all play interdependent roles. Three main types of rejection can occur in a successive manner: (i) hyperacute xenograft rejection, (ii) acute humoral xenograft rejection, and (iii) acute cellular rejection (10). In addition to immunological rejection, coagulation dysregulation, and inflammatory response have become more prominent, leading to xenograft failure.

### Hyperacute Rejection and Acute Humoral Xenograft Rejection

**Figure 1:**
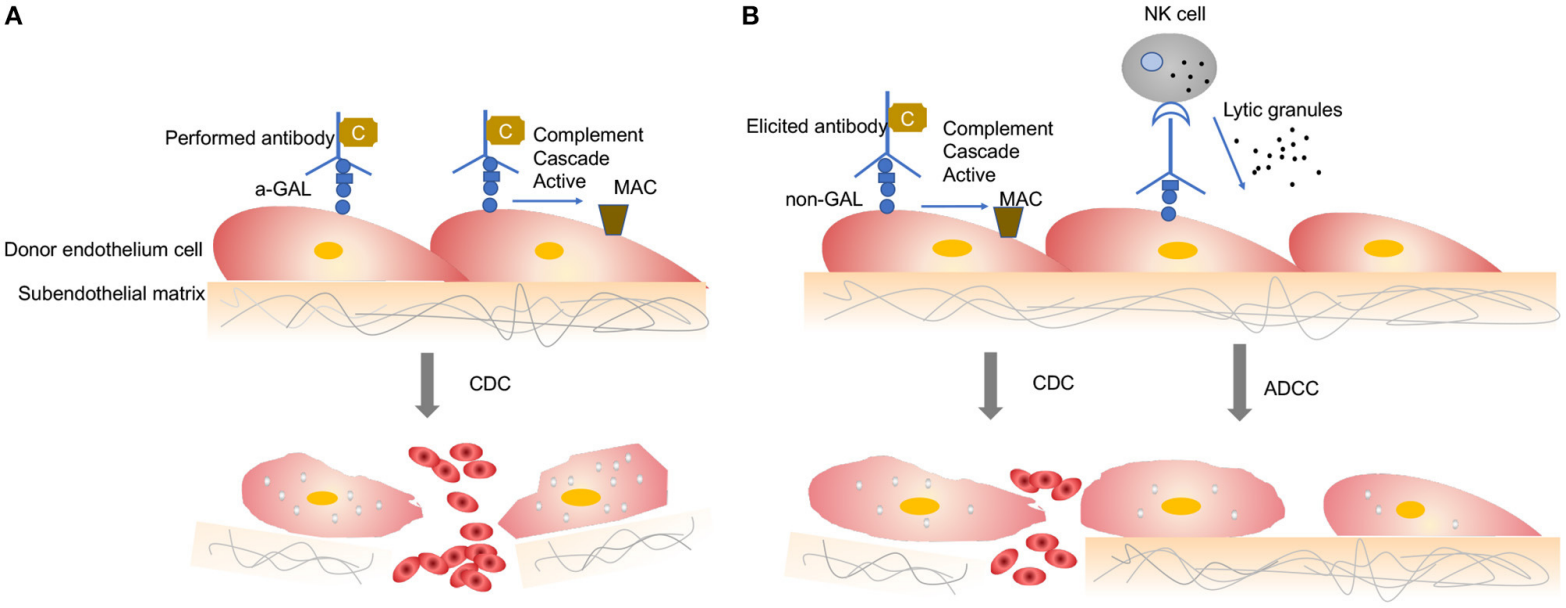
Antibody-mediated xenograft rejection. **(A)** Hyperacute rejection. Hyperacute rejection of vascularized porcine xenografts in untreated primates is triggered by the binding of preformed antibodies to the xenoantigenic epitopes (predominantly α1,3Gal) on the surface of donor endothelial cells. The binding antibody deposition induces the activation of complement proteins and formation of the membrane attack complex, leading to lysis of endothelial cells destruction of the graft vasculature and subsequent graft failure. Loss of endothelial barrier function contributes to bleeding, leading to tissue ischemia and necrosis. **(B)** Acute humoral xenograft rejection. Acute humoral xenograft rejection can be induced by low levels of natural and elicited xenoreactive antibodies. The binding of xenoreactive antibodies to endothelial cells results in complement activation, vascular endothelial activation, and injury caused by antibody-dependent cell-mediated cytotoxicity. Innate immune cells are recruited by activated endothelia and proinflammatory signals. Simultaneously, human antipig antibodies are triggered by natural killer cells and macrophages. MAC, membrane attack complex; C, Complement; NK, natural killers.

When a wild-type pig organ is transplanted into a human or an NHP, the graft is rapidly destroyed, usually within minutes to hours, in a process known as hyperacute rejection (HAR) (11). HAR is a type of humoral rejection and is mediated by preformed antibodies that naturally pre-exist in the recipient. The binding of preformed antibodies to the xenoantigenic epitopes on porcine endothelial cells triggers the activation of complement proteins. Activated complement cause further activation and lysis of endothelial cells, leading to the destruction of the graft vasculature and subsequent graft failure (Figure 1A) (12). HAR is characterized histologically by disruption of vascular integrity, edema, thrombosis, and hemorrhage with widespread vascular deposition of antibodies and terminal complement products (13, 14).

HAR can be avoided by depleting the antipig antibodies or inhibiting complement activation in the recipient by plasmapheresis (15). Although these measures lead to graft survival prolonged beyond 24 h and sometimes for a week or more, the recovered level of antibody has resulted in graft failure, which is known as acute humoral xenograft rejection (AHXR), also referred as “acute vascular rejection” or “delay xenograft rejection” (14). AHXR is a phenomenon caused by a combination of humoral and cellar immune responses, combined with activated endothelia, and inflammation (16, 17) (Figure 1B). Classic features of severe AHXR are massive interstitial hemorrhage, infarction, necrosis, thrombosis, and loss of tubules with polymorph infiltration and massive deposition of immunoglobulin G (IgG), IgM, C3, C4d, and platelets (18). Histopathological features of AHXR are similar to those of HAR (19).

The major HAR xenoantigen is galactose-α1,3-galactose (α-Gal), which is expressed by α1,3-galactosyltransferase (α1,3GalT; also known as GGTA1) (20, 21). GGTA1 is functional in most mammals, including pigs, but not in humans or Old World monkeys (22). In human blood, ~1% of all circulating antibodies are directed against α-Gal epitopes (23, 24). These natural anti-α-Gal antibodies are universally induced during neonatal life by gut bacteria that expressed GGTA1 (25). Pig-to-NHP experiments have shown that Gal-specific antibodies could cause HAR and AHXR (26). Kidneys and hearts from GGTA1 knockout (GTKO) pigs were transplanted into NHP but were rejected through antibody-meditated rejection over several days (18, 27). These data suggest that non-Gal antigens cause AHXR, and non-Gal antigens present an additional barrier to the transplantation of organs from GTKO pigs to humans.

To date, two non-Gal epitopes have been identified: *N*-glycolylneuraminic acid (Neu5Gc) and the SDa blood group (28, 29). The enzyme which is encoded by CMP-N-acetylneuraminic acid hydroxylase (CMAH) gene hydroxylase Neu5Ac to produce the Neu5Gc (30). Humans do not express Neu5Gc because of a DNA mutation that causes them to lack functional CAMH (31), but it is synthesized in some mammals, including pigs and Old World Monkeys (32). Murine deficiency of Neu5Gc and Gal epitopes in xenogeneic cells attenuates the cytotoxicity of naturally occurring antibodies in human sera (33). Evidence *in vitro* suggests that the antibody against Nec5Gc from human serum could bind to porcine Neu5Gc (34). Anti-Neu5Gc antibodies can be induced in humans after dietary intake of porcine tissues from diet (35). The SDa blood group, which is produced by beta-1,4-*N*-acetyl-galactosaminyltransferase 2 (β4GALNT2), is the third examined xenogeneic antigen (29). This antigen was first identified using complementary DNA expression libraries from GGTA1-KO pigs and screening of serum from baboons that had rejected GGTA1-KO pig hearts (36). The inactivation of β4GALNT2 considerably reduces the level of human non-Gal IgM and IgG binding to pig peripheral blood mononuclear cells, suggesting the presence of human antibodies that bind to the porcine glycan produced by the β4GALNT2 gene (34). Therefore, these two non-Gal epitopes may be key barriers to clinical xenotransplantation.

### Cellular Xenograft Rejection

Unlike HAR and AHXR, cellular xenograft rejection is relevant to both whole organ grafts and cellular grafts. It results in rejection that may occur days to weeks after transplantation (37). Cellular rejection of a xenograft can be mediated by innate and adaptive immune responses. These consist of NK cells, macrophages, neutrophils, dendritic cells, T cells, and B cells.

#### Natural Killer Cells in Xenograft Rejection

NK cells are a subset of lymphocytes of the innate immune system. NK cell infiltrates were found in pig organs perfused with human blood *ex vivo* (38, 39) and in pig-to-NHP xenografts (40, 41), suggesting that NK cells participate in xenograft rejection.

**Figure 2:**
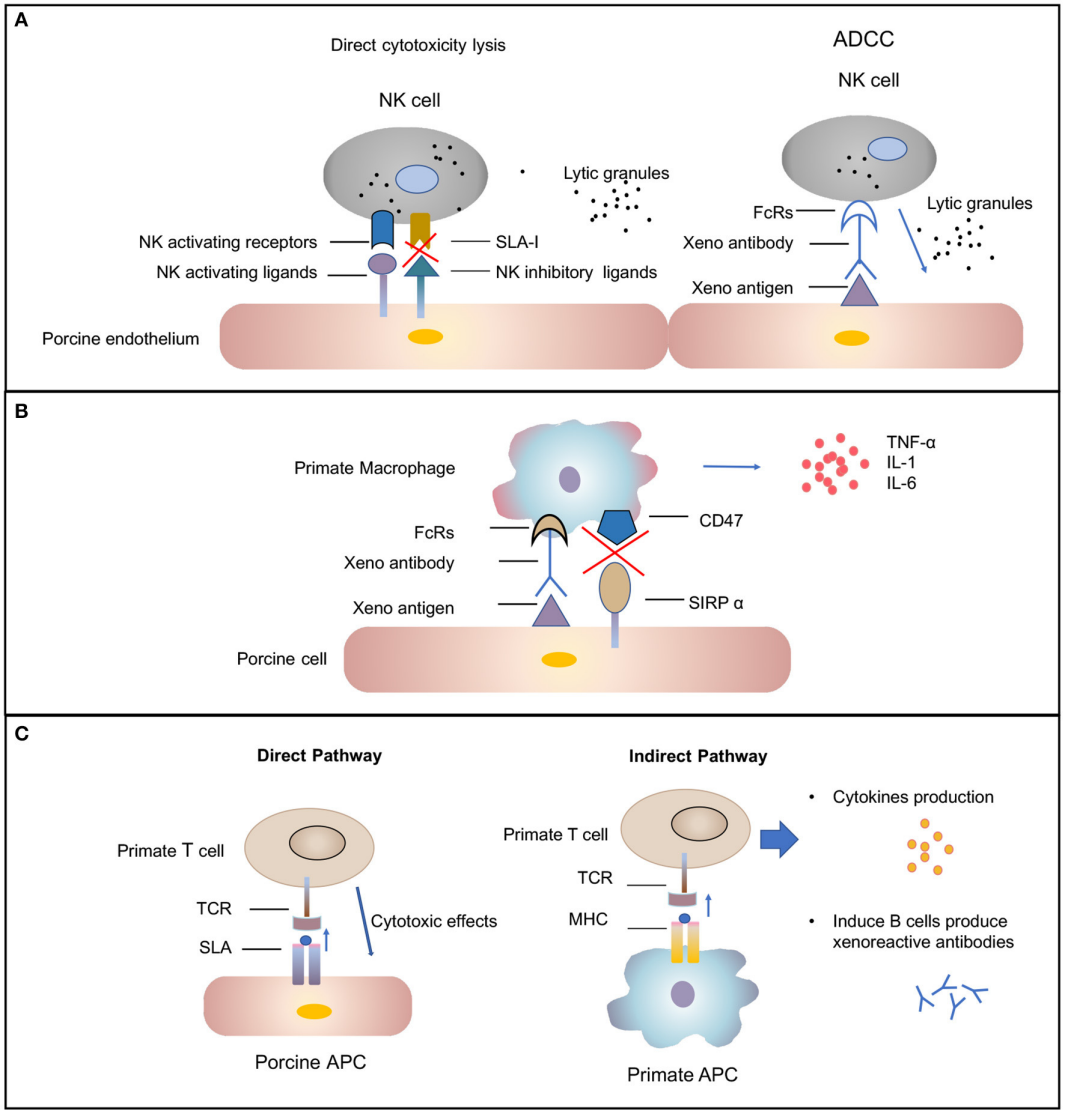
Cellular-mediated rejection. **(A)** Natural killer (NK) cells-mediated rejection. Xenoantibodies bind to donor endothelial cells with their Fab portion. The Fc fraction of the antibody is recognized by FcRs located on the surface of NK cells, triggering the signaling cascade that leads to NK cell destruction. The release of lytic granules (marked as dark spots) leads to pig endothelial cells lysis. The activating NK cell receptors recognize their ligands on the donor cells and trigger lytic granule release. The inability of swine leukocyte antigen (SLA) class I to interact with human inhibitory NK-cell receptors makes porcine cells highly susceptible to human NK-cell-mediated lysis. **(B)** Macrophages-mediated rejection. Macrophages can be activated by cytokines [e.g., interferon gamma (IFN-γ)] that are produced by xenoreactive T cells contributing to the amplification of the T-cell response (not shown). Macrophage also can also be activated by signals mediated by the Fc receptor for IgG (FcγR) upon interaction with xenoreactive-antibody-coated porcine cells. Macrophages secrete proinflammatory cytokines [e.g., tumor necrosis factor alpha (TNF-α), interleukin (IL)-1, and IL-6) that augmented cytotoxicity of macrophages. **(C)** T-cell response in xenograft rejection. The direct pathway refers to the recognition of antigens presented by pig antigen-presenting cells (APCs) by recipient T cells. The T cell is activated by interaction between T-cell receptors (TCRs) and the SLA I and II peptide complexes. This interaction results in T-cell-mediated cytotoxicity that is directed against the xenograft vascular endothelium. The indirect pathway refers to the recognition of donor-derived peptides on recipient APCs by recipient T cells. The interaction between primate TCRs and major histocompatibility complex (MHC) and porcine peptide complexes leads to primate T-cell response, including cytokines production and induction of B-cell activation.

Subsequently the molecular mechanisms involved in human NK cell–porcine endothelial cell interactions have been studied extensively [review in (42)]. Xenograft rejection is mediated by NK cells through direct NK cytotoxicity or by antibody-dependent cellular cytotoxicity mechanisms (Figure 2A). In the direct NK cytotoxicity pathway, through interaction of activating receptors and ligand, NK cells release lytic granules, leading to the lysis of the donor endothelial cell (43, 44). The direct cytotoxicity of NK cell is tightly regulated by the balance between activating and inhibiting signal pathways mediated by a variety of NK cell receptors (45). The activating NK receptors NKG2D (46) and pULBP-1 (47) bind to its pig ligand NKp44 and an unidentified molecule, respectively, to trigger lytic granule release. However, the inhibitory receptors on human NK cell, KIR, ILT2, and CD94/NKG2A, poorly recognize the porcine major histocompatibility complex (MHC) class I molecule, swine leukocyte antigen I, consequently disabling inhibitory signals for NK cell activation (48, 49). The destruction of pig endothelial cells occurs by the recognition of receptors on NK cells. The natural and elicited antibodies deposits on the graft endothelium are recognized by Fc-fraction (FcRs) on NK cells (Figure 2A). Interaction between FcRs and antibodies causes cytotoxic granules to release from NK cells and, in turn, to trigger target cell apoptosis (50). In addition to xenoantibodies bound on the endothelium, the induced antiswine leukocyte antigen (anti-SLA) class I antibodies are recognized by NK cells, also leading to antibody-dependent cellular cytotoxicity (51).

Removal of α-Gal epitopes protects porcine endothelial cells from complement-induced lysis and primate antipig antibodies meditated destruction but does not resolve the adhesion of NK cells and direct NK cytotoxicity (52). These data suggest that α-Gal residues on porcine cells may not be involved in the increased adhesion and direct cytotoxic activity of human NK cells.

The role of NK cells in xenotransplantation still must be fully elucidated. The majority of knowledge on NK in xenotransplantation was generated for *in vitro* studies and in pig to rodent models. Further *in vivo* studies on NHPs are required for a better understanding of the role of NK cells in the rejection of porcine cellular and organ xenografts.

#### Macrophage Cells in Xenograft Rejection

Macrophages have been found to be involved in the rejection of both organ grafts and cellular grafts (53). A dense macrophage infiltrate was identified in all the rejected xenografts through histologic analysis (54). Macrophage contribute to xenograft rejection by their activity of modulation adaptive immunity and direct cytotoxicity (37). Macrophages activity can be result from xenoreactive T cells. T cells recruit and activate macrophages, causing infiltration, and the destruction of xenografts by macrophages. This process, in turn, leads to T-cell response amplification (55, 56). In addition, macrophages can be active by direct interaction between donor endothelial antigens and receptors on the surface of macrophage (Figure 2B) (57). Macrophages perform direct toxic effects mediated by the production of proinflammatory cytokines [e.g., tumor necrosis factor alpha (TNF-α), interleukin-1 (IL-1), and IL-6] that are secreted by macrophages (58). Therefore, the regulation of macrophage activation should improve xenograft survival.

A number of inhibitory receptors have been reported to inhibit phagocytic activity. Among many pathways, the signaling regulatory protein (SIRP-α)–CD47 signaling pathway is an important negative pathway to macrophages. SIRP-α recognizes CD47 as a marker of self, preventing macrophage-mediated autologous phagocytosis (59, 60). Research has indicated that interspecies incompatibility between CD47 and SIRP-α contributes to the rejection of xenogeneic cells by macrophages (61) and that binding porcine CD47 does not supply the inhibiting signal through SIRP-α to human macrophages (62). Other inhibitory molecule, such as CD200 (63), immunoglobulin-like transcript 3 (64), and Ig-like receptor B (65), have been reported involved in macrophage function. However, whether incompatibility between these molecules on pig cells and their receptors on primate macrophages promotes macrophage activation in xenogeneic immune responses requires further evaluation.

#### T-Cell Response

T lymphocytes are likely important mediators of acute cellular rejection. Similar to allotransplantation, T cells are activated through both direct and indirect pathways after xenotransplantation (Figure 2C) (66). In the direct pathway, pig antigen-presenting cells (APCs) directly active primate T cells. The interaction between primate T-cell receptors and SLA class I and II peptide complexes results in T-cell-mediated cytotoxicity against the xenograft vascular endothelium. The two cell types likely to be donor APCs are the migratory passenger leukocytes and porcine endothelial cells constitutively expressing CD80/86 (67). In the indirect pathway, T cells activation occurs through donor-derived peptides presented by recipient APCs. Pig xenoantigens are recognized by MHC class II of the recipient and presented to host T cells (66). This process, in turn, leads to CD4^+^ T-cell stimulation, B-cell activation, *de novo* antibody production, and humoral xenograft rejection. The cytotoxicity of NK cells and macrophages also can be substantially augmented by cytokines produced by xenoantigen-activated T cells (12).

Although similar immunological mechanisms can be observed in allotransplantation and xenotransplantation, T-cell responses against pig antigen, especially in indirect responses, are stronger than responses against alloantigen (68). Surprisingly, acute cellular rejection, as seen in the majority of allotransplants, is rarely documented after pig-to-NHP organ xenotransplantation. There are two possible reasons for this result: either humoral rejection is so strong that we cannot observe cell rejection following xenotransplantation, or current immunosuppression therapy is sufficient to control T-cell-mediated response in xenotransplantation (69). T-cell activation requires the binding of the TCR to an MHC–peptide complex on the APC as well as a second costimulatory signal involved in the CD40–CD154 and CD28–CD80/86 pathways (70). The compatibility of cross-species adhesion and costimulation molecules is a critical issue in a xenogeneic context. The strategies to alleviate T-cell rejection in xenografts rely mainly on promoting costimulation and downregulation of MHC expression in porcine cells. In 2000, costimulation blockade-based immunosuppressive therapy was introduced into xenotransplantation by Buhler et al. (71). The initial agent, anti-CD154mAb, was highly effective at preventing T-cell response in the pig-to-NHP model (72). Unfortunately, anti-CD154mAb was found to be thrombogenic and is currently not available for clinical use (73). ln subsequent research, anti-CD40mAb, which also blocks the CD40–CD154 pathway, was found to be equally effective in xenotransplantation (74, 75). However, currently available anti-CD28 agent alone may be insufficient prevent a T-cell response in NHP models (76). Taken together, the blockade of the CD40–CD154 pathway is a critical component of immunosuppressive agents in the control of xenogeneic T-cell response.

### Coagulation Dysregulation

When HAR, AHXR, and T-cell response are prevented, coagulation dysregulation becomes more obvious following xenograft transplantation and is considered another major barrier to prolonged xenograft survival in NHPs (69). Coagulation dysregulation results in the development of thrombotic microangiopathy in the graft. Features of thrombotic microangiopathy include fibrin deposition and platelet aggregation resulting in thrombosis within the vessels of the graft and eventual ischemic injury (77, 78). With the development of coagulation dysregulation, systemic consumptive coagulopathy may be observed in the recipient and lead to the recipient's death, but this phenotype does not occur in all xenograft organs (79).

**Figure 3:**
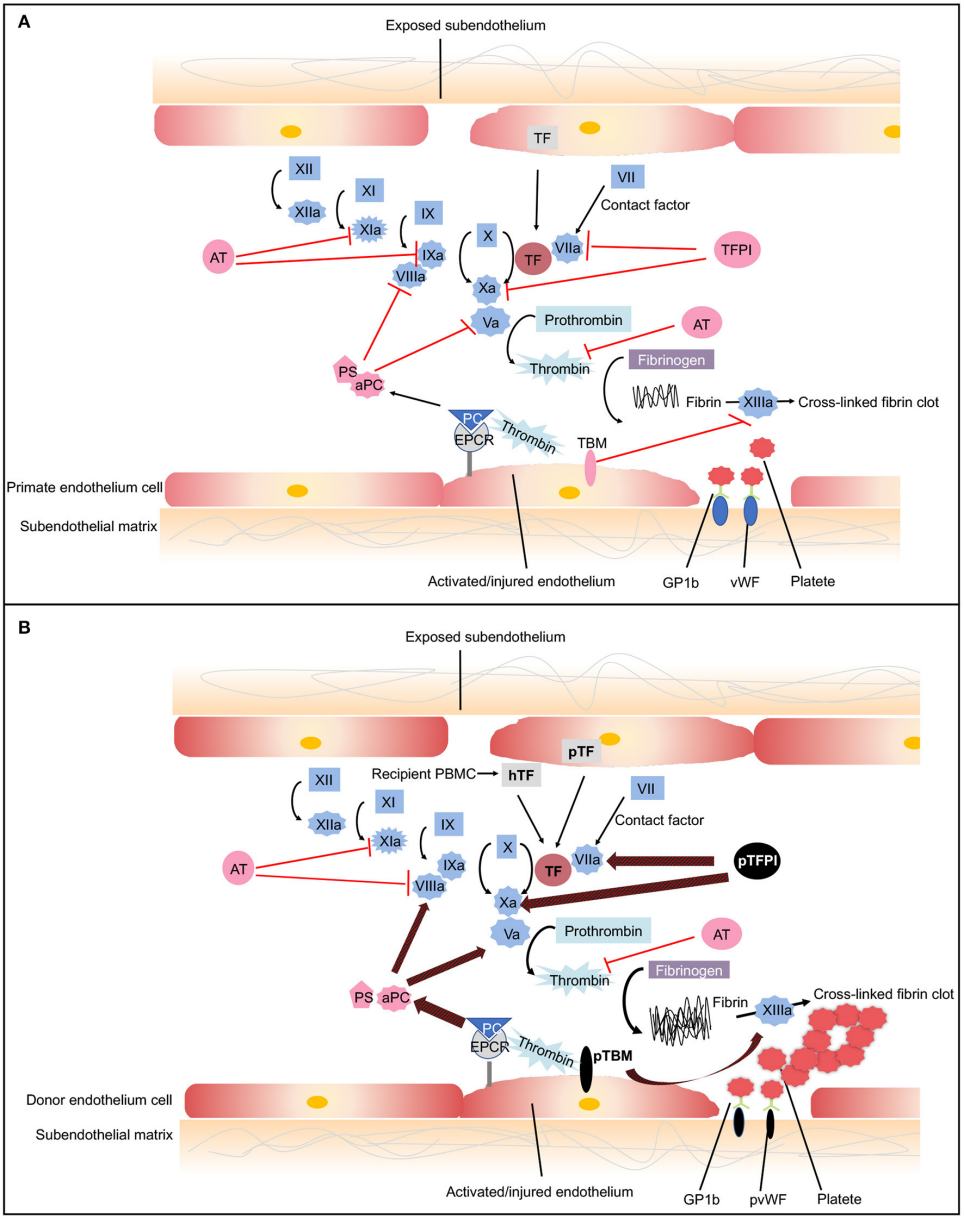
The coagulation cascade related to xenotransplantation. **(A)** Coagulation cascade in primates. Black arrows designate cascade amplification steps. The coagulation cascade is initiated by tissue factor (TF) (extrinsic pathway) or negatively charged surface contact (intrinsic pathway). TF is expressed by vascular subendothelial cells. When endothelium is damaged, TF is exposed to the circulation and forms complexes with factor VIIa, activating factors V and X. Factor Xa converts prothrombin to thrombin. Thrombin then cleaves fibrinogen into fibrin monomers and activates factor XIII, which cross-links fibrin monomers into an insoluble clot. In response to shear stress, von Willebrand Factor (vWF) binds to glycoprotein 1b (GPIb) on platelets leading to platelet activation and adhesion (83). Activated platelet bind to fibrinogen to mediate platelet aggregation and endothelial adherence. Red lines show the natural inhibitors of coagulation. (1) Tissue factor pathway inhibitor (TFPI) inhibits the activation of factor Xa and formats TFPI/Xa, which subsequently inhibits the TF/VIIa complex. These processes consequently prevent the formation of thrombin (81). (2) In the thrombomodulin (TBM)–protein C (PC) pathway, TBM serves as a cofactor in the thrombin-induced activation of PC. Endothelial protein C receptor (EPCR) is a receptor for PC that enhances its activation. The activated PC (aPC), together with cofactor protein S (PS), suppressing factors Va and VIIIa, thereby downregulating thrombin formation and suppressing coagulation cascade (84). (3) Soluble antithrombins (AT) inhibits factors XIa, IXa, Xa, VIIa, and thrombin by targeting serine proteases (82). **(B)** Dysregulated coagulation in pig-to-primate xenotransplantation. Red and black arrows designate incompatibility between pig and primates. When pig endothelium is activated, pig TF is expressed and released into the circulation. After interaction with the pig endothelium, recipient platelets and peripheral blood mononuclear cells (PBMCs) express primate tissue factor (hTF). The porcine TF (pTF) pathway inhibitor is an ineffective inhibitor of the human Xa factor and may ineffectively shut down the activation of the major TF. Pig TBM (pTBM) binds only weakly to primate thrombin, leading to levels of activated PC that are insufficient to inhibit coagulation, resulting in thrombotic microangiopathy in pig grafts within a matter of weeks (85). Porcine vWF spontaneously could aggregate primate platelets through GPIb receptors even in the absence of shear stress (86). After aberrant porcine GPIb–primate vWF interaction, platelets are activated. Small vessels in the graft become occluded by fibrin and platelet aggregation.

Coagulation is a complex pathway that involves interactions with inflammation and innate immunity (80). Normally, coagulation occurs continuously within the bloodstream but is restrained by anticoagulants, thus maintaining coagulation balance (81). When endothelial cells are injured, tissue factor (TF) is liberated into circulation, triggering the extrinsic coagulation pathway. The increased coagulation is initiated by TF, which forms complexes with factor VIIa. The coagulation cascade then becomes amplified by the factors shown (VIIa/TF complex, IXa, and Xa), which in turn activate thrombin (81). A network of inhibitory pathways, including tissue factor pathway inhibitor (TFPI) and thrombomodulin (TBM)–protein C (PC) pathway, regulate the coagulation balance (82) (Figure 3A).

In the context of xenotransplantation, the assault by antibodies and complement-activated pig endothelial cells converts endothelial cells from an anticoagulant phenotype to a procoagulant state, leading to vascular destruction, and infiltration by various immune cells (87). Both recipient- and donor-derived TF contribute to activation of the extrinsic coagulation cascade (85, 88). The molecular incompatibilities between primate and pig coagulation–anticoagulation systems exaggerate this process (Figure 3B). The porcine TFPI is not sufficient to inhibit factor Xa of primates and ineffectively shuts down the activation of the major TF (89). Porcine TBM also fails to regulate primate thrombin. Porcine TBM binds human thrombin less strongly and thus does not activate PC (90). Another molecular incompatibility is porcine von Willebrand Factor (pvWF) and primate platelet lycoprotein 1b (GPIb). Even in the absence of shear stress, pvWF spontaneously aggregates primate platelets through GPIb receptors (86). After aberrant GPIb–vWF interaction, intracellular signaling occurs, and platelets are activated. Activated platelets develop thrombosis after being recruited to the place of the endothelial cells' injury, which leads to widespread activation of the coagulation system (91).

Above all, recent advances in the field of xenotransplantation have enabled a better understanding of the immune mechanisms underlying the failure of porcine xenografts. It is vital for xenotransplantation be introduced into clinic. However, many molecular mechanisms underlying xenograft rejection needed further elucidation, especially in pig-to-NHP models. Apparently, considerable “cross-talk” occurs between the cellular and humoral immunology responses and between those responses and the factors responsible for coagulation dysfunction and inflammation in rejected xenografts. As a consequence, diverse strategies are required to overcome the various immunological barriers involved in the rejection of various forms of xenotransplantation procedures.

## Genetically Modified Pigs for Xenotransplantation

According to studies on immunological rejection and coagulation dysregulation, plenty of genetically modified pigs were generated to bridge cross-species molecular incompatibilities. Since 2009, most of the advances that have been made in the field of xenotransplantation because of the production of genetically engineered pigs. As a result of improvements in gene-editing tools, especially clustered regularly interspaced short palindromic repeats-cas9 (CRIPSR/Cas9), a large variety of genetically modified pigs have been generated, and the production of source pigs with multiple edited genes has become easier and faster (92). In this section, we summarize current genetically modified pigs available for xenotransplantation (Table 1).

**Table 1:** Genetically modified pigs currently available for xenotransplantation research.

**Abbreviation**	**Gene name**	**Function**	**Reference**
GTKO	1,3-galactosyltransferase KO (GGTA1 KO)	Deletion of αGal epitope	(93)
CMAH KO	CMP-*N*-acetylneuraminic acid hydroxylase KO	Deletion of Neu5Gc epitope	(94)
β4GalNT2 KO	β-1,4*N*-acetylgalactosaminyltransferase KO	Deletion of SDa epitope	(34)
hCD46 (MCP)	Human membrane cofactor protein transgene	Inactivation complement factors C3b and C4b	(95)
hCD55 (DAF)	Human decay accelerating factor transgene	Acceleration of complement decay	(96)
hCD59 (MAC-IP)	Human membrane attack complex C5b-9 inhibitory protein transgene	Inhibition of the complement membrane attack complex C5b-9	(97)
hTBM	Human thrombomodulin	Anticoagulation (activates protein C)	(98)
hTFPI	Human tissue factor pathway inhibitor	Antagonize the function of tissue factor	(99)
hCD39 (hENTPD1)	Human ectonucleoside triphosphate diphosphohydrolase-1 transgene	Anticoagulation and anti-inflammatory	(100)
hA20	Human tumor necrosis factor alpha-induced protein-3 transgene	Inhibition of NF-kappa B activation and TNF-mediated apoptosis	(101)
hCD47	Human integrin associated protein transgene	Regulation of macrophage activation and phagocytosis	(102)
CTLA4-Ig	Cytotoxic T-lymphocyte-associated protein 4-immunoglobulin transgene	Cellular immune response: Inhibition of T-cell costimulation via CD86/CD80	(103, 104)
CIITA-DN	MHC class II transactivator dominant negative	Suppression of T-cell activation	(105)
hHO1	Human heme oxygenase 1 transgene	Antiapoptosis; cytoprotection; anti-inflammatory	(106)
ASGR1 KO	Asialoglycoprotein receptor 1	Decreases human platelet phagocytosis by pig sinusoidal endothelial cells	(107)
PERV inactivation	Porcine endogenous retroviral virus inactivation	Xenozoonosis	(108)

### Expression of Human Complement Regulatory Proteins

Complement activation is a clearly detriment factor in contributing to xenograft failure. One approach is administering an agent to inhibit complement, but such treatment only had a temporary effect and enhanced the risk of infection (15, 109). Another approach is engineering genetically modified pigs to overcome immunological rejection. Pigs possess complement regulatory proteins (CRPs) that are similar to those of humans, but pig CRPs are not sufficient to protect pig epithelium cells from human complement-mediated injury. Introduction of human CRPs (hCRPs) (e.g., CD46, CD59, and CD55) into pig cells was suggested to inhibit complement-mediated graft injury. In the 1990s, two independent research groups first proposed the suggestion that production of transgenic pigs expressing the human CRPs CD59 (110) and CD55 (111) to protect from hyperacute xenograft rejection. Then, pigs expressing hCRP were produced by microinjection of DNA into the fertilized egg (96, 97). These advances introduced the possibility of genetic modification of the organ-source pig for xenotransplantation. Today, many pigs expressing hCPRs have been produced [reviewed in (112)]. Researches have also demonstrated that expression of hCRPs can inhibit complement-mediated graft injury and prolong xenograft survival time (113, 114). Furthermore, studies have also demonstrated that a combination of hCRPs offers greater protection than the expression of just one hCRP (115, 116).

### Deleting Xenoreactive Antigens

Rejection of anti-Gal antibodies can be prevented through plasmapheresis (117) or using immunoaffinity columns (118). However, these approaches have demonstrated only partial success because the graft is lost when antibody levels recover. Pigs with heterozygous GTKO using homologous recombination were produced in 2002 (119, 120), and homozygous GTKO pigs were produced in 2003 (93, 121). Initial studies indicated that GTKO pigs protect xenografts from injury as a result of HAR after heart and kidney transplants to NHPs (78, 122). The production of GTKO donor pigs is a milestone of xenotransplantation field.

In addition to GTKO pigs, genetically modified pigs with GGTA1/CMAH (123) or GGTA1/ B4GalNT2 (124) knockout (DKO) and GGTA1/CMAH/B4GalNT2 triple knockout (TKO) (125) were also produced. A study demonstrated that cells from GGTA1/CMAH DKO pigs bound a reduced human antibody than GTKO pigs (126). Moreover, xenografts from GGTA1/CMAH DKO pigs reduced the consumption of human platelets in the liver model (127). These results suggest that the deletion of Neu5Gc epitope in pigs is crucial for increasing xenograft survival time. *In vitro* evidence has also suggested that inactivation of the B4GalNT2 gene reduce human antibody binding (34, 128). These data indicate that TKO pig organs have proven to be a major advancement compared with GTKO and DKO xenografts following transplantation into human. However, experiments involving xenografts from TKO pigs to NHPs have not been reported, mostly because Old World monkeys express Neu5Gc (129). By contrast, New World monkeys do not express Neu5Gc and are likely to produce anti-Neu5Gc antibodies (130). Therefore, this animal is the preferred candidate model for evaluating the effect of the Neu5Gc deletion xenograft in NHP models.

Today, the importance of α-Gal in immunological rejection is clear, GTKO pigs are considered to be the basis for further genetic modifications. Studies have demonstrated that the transplantation of organs from GTKO/hCRP pigs has a more favorable effect than the transplantation of organs from pigs with GTKO or CRP alone (131, 132). Although expression of hCRP alone does not enable make graft long-term survival, even in the GTKO pigs, the complement system is still activated by ischemia–reperfusion injury. Herein, the deletion of identified xenoantigens with expression of one or more hCRPs in pigs would form the foundation for future clinical trial.

### Inhibition of Cellular Xenograft Rejection

Because of the aforementioned incompatibility between human SIRP-α and pig CD47, genetically engineered pigs expressing human CD47 (hCD47) have the potential to resolve this problem. *In vitro*, porcine cells expressing hCD47 can reduce their phagocytosis by human macrophages (62). Moreover, the expression of hCD47 in porcine endothelial cells not only suppressed macrophage-mediated cytotoxic activity and inflammatory cytokine (TNF-α, IL-1β, IL-6) production but also inhibited human T-cell infiltration (133). *In vivo*, hCD47 expression increased xenogeneic hematopoietic engraftment chimerism in the murine model (102) and prolonged the survival porcine skin grafts in baboons (134). These findings collectively suggest the beneficial role of hCD47 expression in xenografts. However, hCD47 expression did not completely prevent phagocytosis from primate macrophages; therefore, the pathway of xenoantigen-activated macrophages may also need to be suppressed.

Cytotoxic T-lymphocyte-associated antigen (CTLA4) is a costimulatory molecule that blocking B7-CD28 costimulatory pathway (135). Immunosuppression therapy with human CTLA4-immunoglobulin (hCTLA4-Ig) has been shown to extend graft survival during allotransplantation for NHPs (136). Therefore, transgenic pig expression of hCTLA4-Ig is a potential means of preventing T-cell activity. Martin et al. produced transgenic pigs with neuronal expression of hCTLA4-Ig and demonstrated that hCTLA4-Ig protein reduced the proliferation of human T cells against porcine cells (103). The beneficial effect of hCTL4-Ig expression extended xenograft survival time in a rat skin transplantation model (104) and a NHP neuronal transplantation model (137). These *in vivo* evidence also suggested that the expression of hCTLA4-Ig alone could not prevent xenograft rejection, which is consistent with the result blocking costimulatory pathway against B7-CD28 only. Transgenic pigs ubiquitously expressing pCTLA4-Ig were also produced (138). However, these pigs were susceptible to infection because of high levels of pCTLA4-Ig expression in the blood. Therefore, the expression of this agent only in specific target cells of the pig is favorable. In addition, T-cell response can be controlled through the deletion of SLA class I (139) or introduction of a mutant human class II transactivator gene (CIITA-DN) in pigs (105), both of which reduce pig antigen presentation in the direct pathway. However, the role of these modified genes in protecting xenograft from rejection response requires further evaluation in NHPs. Moreover, although not the original intention, lacking α-Gal antigens or expression of hCRPs has been demonstrated to reduce T-cell response to pig cells (140, 141).

### Expression of Human Coagulation Regulation Proteins

Using GTKO/hCRP genetically modified pigs as the source donor, HAR and AHXR have been controlled well in many pig-to-NHP model studies (72, 74, 142). However, both thrombotic microangiopathy and systemic consumptive coagulopathy are increasingly recognized in xenograft and NHP recipients. Therefore, coagulation dysregulation becomes a non-negligible barrier to successful xenotransplantation. The graft vascular endothelial cells enter into a procoagulant state, which cannot be successfully controlled by the pig's anticoagulant factors, resulting in coagulation dysregulation and graft failure. This problem may be resolved by further overexpression of human coagulation regulation proteins, such as TBM, endothelial protein C receptor (EPCR), TFPI, and CD39 in the organ-source animals.

Transgenic expression of hTBM in donor pig is one of most important approaches to overcoming coagulopathy currently. Pig aortic endothelial cells expressing hTBM were reported to substantially suppress prothrombinase activity, delay human plasma clotting time, and exhibit less activity in inducing human platelet aggregation (143, 144). In the pig-to-baboon model, hTBM expression on cardiac xenografts confers an independent protective effect for prolonging graft survival time (145, 146). Another key player in the anticoagulation system is EPCR, which also mediates anti-inflammatory and cytoprotective signaling (147). Therefore, it is speculated that overexpression of hEPCR in donor pigs is a potential solution to overcoming related barriers, providing potent local anti-inflammatory, anticoagulant, and cytoprotective cell signaling. *In vitro*, cells from GTKO/CD46 pigs that also expressed EPCR reduced platelet aggregation activity (144). Expression of hTFPI is a potential approach to resolving the incompatibility between human TF and pig TFPI. *In vitro* study demonstrated that expression of hTFPI can inhibit TF activity (99). CD39 plays a key role in the regulation of coagulation. CD39, which is responsible for catalyzing the degradation of extracellular ATP, ADP, and AMP, can inhibit thrombus formation. One study demonstrated that transgenic hCD39 expression in pigs protected against myocardial ischemia/reperfusion injury in an *in vivo* model (100). In addition, vWF-deficient donor pigs exhibited prolonged lung graft survival time in NHP models and caused a less substantial platelet decrease in receipts (148, 149).

Graft coagulation varies among different xenograft organs after transplantation, perhaps because of differences in vascular structure and protein expression pattern. Recently, considerable progress has been made in cardiac and renal xenotransplantation. However, improvements have been limited in liver and lung xenotransplantation. After pig liver xenotransplantation, severe thrombocytopenia can occur within minutes to hours, which exacerbates coagulation dysfunction, resulting in lethal hemorrhage (150). PvWF is a glycoprotein that plays a key role in the pathogenesis of xenograft failure, especially in pulmonary xenotransplantation, because the lung releases more vWF than the heart or kidneys (151). Moreover, the transcription of genes involved in coagulation, fibrinolysis, and platelet function differs in heart and kidney xenografts, which may account for the different courses of coagulation dysregulation in the recipients of these organs (152). Pulmonary xenografts release larger quantities of vWF than do heart and kidney xenografts (148). Meta-analyses suggest that the most influential factors in lung transplantation are EPCR and CD39 rather than TBM and TFPI (153). These data collectively suggest that successful control of coagulation dysregulation in xenotransplantation may require different genetic and pharmacological strategies for different organs.

### Expression of Human Anti-inflammatory Proteins

An increasing amount of evidence suggests that inflammatory response plays a considerable role in graft failure in cases of a condition called systemic inflammatory response in xenograft recipients (154). Therefore, the engineering of donor pigs that express one or more human anti-inflammatory or antiapoptotic genes may be an approach to xenograft protection. Transgenic pigs that express human hemeoxygenase-1 and human A20 are available (101, 106). The expression of human hemeoxygenase-1 reportedly protected porcine kidneys from xenograft rejection in the case of *ex vivo* perfusion with human blood and transgenic porcine aortic endothelial cells (106). However, several human transgenes, including hHO1, hCD47, and human A20, have been introduced in pigs with multiple genetic manipulations (155).

As discussed above, numbers of genes have been found to be involved in xenograft rejection. Because of the immune response to a pig xenograft cannot be considered in isolation, successful control of immunological rejection in xenotransplantation requires the altering of multiple genes in donor pigs. Genetically modified pigs with multiple genes, with up to seven manipulations, have been produced. The *in vivo* evaluation of their individual specific benefits will be difficult, and it remains unknown whether the manipulation of so many genes in donor pigs has adverse effects. Therefore, the optimization of combinations of modified genes in donor pigs and evaluation of these xenografts in NHP models are important in further studies.

## Pig Organ Graft Survival in Non-human Primates

Xenotransplantation has a long history with a number of animal models, including mouse, rat, and NHP, and has been used to reveal the mechanisms of rejection responses (156, 157). Old World NHPs are the preferred surrogate for humans in exploring the response to pig xenograft transplantation because of their immunological similarities to humans (6). Today, the pig-to-NHP model is the standard model for testing the primate immune response to organs or tissue from genetically modified pigs and the effect of novel immunosuppressive regimens. It is considered the optimal testing ground for predicting human responses as the final step before a human clinical trial (158). Two comprehensive reviews explored pig solid organ graft survival in an NHP until 2013 (159, 160). More recently, several studies reported key advances in NHP models. In this section, we summarize the studies of solid organs in preclinical models in recent years (Table 2).

**Table 2:** Best survival time of solid organ xenotransplantation from pigs to non-human primates.

**Xenograft**	**Donor pigs genetic background**	**Immunosuppressive therapy**	**Survival time (day)**	**Year (Reference)**	**Initial survival time (donor pig)**
Heart (non-life supporting)	GTKO/CD46/TBM	ATG, anti-CD40mAb, anti-CD20mAb, MMF, CVF, Solu-Medrol, aspirin, heparin, Ganciclovir, Cefazolin, Epogen	Rang from 159 to **945** Median: 298 days *n* = 5	(145)	6 h (WT)
Heart (life supporting)	GTKO/CD46/TBM	Anti-CD20mAb, ATG, anti-CD40mAb, MMF, methylpednisolone, temsirolimus, steroid cortisone	18, 27, 40, **195** *n* = 5	(161)	9 days (hCD55)
Kidney (life supporting)	GTKO/CD55	Anti-CD154 mAb, MMF, solumedrol	**499**, 414, >70 *n* = 3	(162)	13 days (WT)
Lung	GTKO/CD47/CD55	ATG, rituximab, anti-CD154 mAb and mycophenolate mofetil	**14**, 13, 4, 2, 1 *n* = 5	(163)	11 h (WT)
Liver	GTKO	ATG, anti-CD40mAb, tracrolimus, Cs, CVF, hPCC	25, **29** *n* = 2	(164)	84 h (WT)

*ATG, antithymocyte globulin; MMF, mycophenolate mofetil; CVF, cobra venom factor; hPCC, human prothrombin complex concentrate*.

*Bold values represent the longest survival time of xenograft*.

### Heart Xenotransplantation

Most studies on pig heart transplantation in NHPs have been heterotopic. The survival time of the graft was only 4–6 h following transplant with a wild-type pig heart (165). Since GTKO pigs were introduced in 2003, GTKO only or donor pigs expressing one or more hCRPs have helped to prolong the xenograft survival time [reviewed in (160)]. In 2012, Mohiuddin et al. transplanted GTKO/hCD46 transgenic pig hearts into baboons administered anti-CD154 mAb-based immunosuppression (166). They extended the longest survival for heterotopic cardiac xenografts to 236 days. However, thrombotic microangiopathy was observed in the xenograft, and coagulation dysregulation is likely to be the major obstacle in achieving longer survival rates. Subsequently, same authors used GTKO/hCD46/hTBM donor pigs combined with a CD40 antibody-based immunomodulatory regimen (2C10R4) for heterotopic heart transplantation in pig to NHP models (145). In their experiments, the longest survival time was extended to 945 days with a median survival of 298 days. Furthermore, none of the subjects experienced consumptive coagulopathy or thrombocytopenia. This study demonstrated the efficacy and safety of a CD40 antibody-based immunomodulatory regimen (2C10R4) in recipients and suggested the important role of hTBM in donor pigs.

Although considerable progress has been made in non-life supporting heart xenotransplantation, the life-supporting heart xenotransplantation is still difficult in NHPs; moreover, it is also vital to justify the potential clinical application of heart xenotransplantation. Until 2018, the longest survival time of life-supporting heart transplantation in pig-to-NHP cases was only 57 days (167). On the bases of previous studies, Langin et al. modified their procedure and reported a survival time of more than 6 months in cases of life-supporting heart xenotransplantation in baboons (161). In their protocol of heart xenotransplantation, two steps were crucial to prolong the survival of functional xenografts in baboons. First, non-ischemic porcine heart preservation was performed instead of cold static storage. Second, detrimental xenograft overgrowth was restricted by a drug called temsirolimus (Table 2). The immunosuppression protocol used in this study seems to have been tolerated by the baboons because of no major immunosuppression-related infection observed. Their encouraging data suggest that their method might be safe for use in humans, and their research constitutes vital progress toward making clinical heart xenotransplantation a reality.

### Kidney Xenotransplantation

Although the kidney is transplanted as a vital organ, progress in the use of kidneys in pig-to-NHP models has been slower than that for the use of the heart. Before 2015, life-sustaining pig kidney xenotransplantation was limited to only a few weeks on average, with the longest reported survival in pig-to-NHP models being 90 days [review in (160)]. In 2015, GTKO/hCD55 pigs was used as donors and rhesus macaques with T-cell deletion as recipients with follow-up maintenance therapy of anti-CD154 mAb. Recipients with lower titers of antipig antibodies exhibited prolonged kidney xenograft survival (more than 125 days) (72). Compared with previous reports, features of consumptive coagulopathy and proteinuria were delayed for many months. Iwase et al. also reported the transplantation of a kidney from a GTKO/CD46/CD55/TBM/EPCR/CD39 pig (TBM and CD39 were very poorly expressed in the kidney) to a baboon treated with an anti-CD40mAb-based regimen, and the kidney functioned for 136 days (168). This study suggested that the anti-CD40mAb-based regimen was likely to be of equal benefit to anti-CD154 mAb; it also noted the potential beneficial effects of anti-inflammatory agent. In their later study, kidneys from GTKO/CD46/CD55/EPCR/TFPI/CD47 pigs functioned normally in the baboons until days 237 and 260. Two baboons died from infection rather than from immune rejection, and no features of consumptive coagulopathy were observed (169). They suggested that the expression of EPCR is critical to prevent kidney xenograft from coagulation dysregulation.

In 2018, kidneys from GGTA1/B4GALNT2 DKO pigs were transplanted into rhesus monkeys who were immunosuppressed with T-cell depletion, anti-CD154, mycophenolic acid, and steroids. The longest survival achieved in these recipients with functioning transplants was 435 days (124). However, analysis of xenografted kidneys revealed that antibody-meditated rejection and coagulation dysregulation are still the causes of graft failure. Additional deletion of xenoantigen and introduction of human anticoagulation gene would be required in kidney xenografts. More recently, Kim et al. reported their newest report in pig to rhesus macaque kidney transplantation with the longest survival of a life sustaining xenograft in an NHP (499 days) and consistent survival over 1 year (162). Based on previous study of their group (72, 169), GTKO/CD55 pigs were used as donors and rhesus macaques with CD4^+^ T-cell deletion and lower titers of antipig antibodies were as recipients. This is the first translation model of life-sustaining kidney xenotransplantation, achieving the longest survival time for pig kidney xenografts in NHP models to date. This study determined that the depletion of the CD4+ T cell before transplantation is necessary for the long-term survival of the xenograft. However, the mechanism that selective CD4+ T-cells depletion was sufficient to protect xenograft remains unknown. Whether additional modification such as SLA class II knockout is necessary for donor pigs requires further investigation.

Another question in kidney xenograft is hypoalbuminemia, which developed from proteinuria and uniformly documented in the early studies. However, only modest proteinuria without accompanying hypoalbuminemia has been observed in NHPs with pig kidney grafts recently. More effective control of immunological rejection by genetically modified pig and immunosuppressive agents might be beneficial for this problem.

### Liver Xenotransplantation

Pig liver xenotransplantation seems to be more difficult to perform compared with heart and kidney xenotransplantation. The relevant molecular mechanisms of xenogeneic rejection involved in liver xenografts are more complex. After liver xenotransplantation, thrombotic microangiopathy in the graft and systemic consumptive coagulopathy appear to be more severe after pig liver xenotransplantation (170).

The first report of pig liver orthotopic xenotransplantation to NHPs dates back to 1968, at which time immunosuppression was limited and donor pigs were wild type, resulting in a maximum survival of only 3.5 days (171). Since 2010, GTKO and GTKO/hCD46 pigs have been introduced for liver xenotransplantation (172–174). Livers from genetically modified pigs were transplanted into baboons, extending liver graft survival time up to 9 days. The limited survival time of liver xenograft was predominantly due to the development of a lethal coagulopathy. Recently, the survival time for life-supporting orthotopic GTKO pig liver xenografts was extended to 25 and 29 days with hepatic function in baboons, which represents the longest survival time following pig-to-primate liver xenotransplantation to date (164, 175). In their modified experimental protocol, the addition of a costimulation blockade agent, anti-CD40 mAb, was ostensibly critical to prolonging liver survival. Moreover, baboons were treated using a continuous infusion of human prothrombin concentrate complex, an exogenous human coagulation factor, to prevent coagulation dysregulation and allow spontaneous platelet count recovery.

### Lung Xenotransplantation

The pig lung is the organ most severely damaged by rapid coagulation dysfunction (176). Most research on lung transplantation has employed *ex vivo* pig lung xenoperfusion with human donor blood models (177), but this model is limited to only short-term effects, usually those occurring within 4 h (178). Lung xenotransplantation research has begun to use pig-to-NHP models. Nguyen et al. demonstrated that the lungs of GTKO pigs with life support transplanted into baboons were protected from HAR. However, the xenografts were functional for only 3.5 h because of severe coagulation dysregulation (179). Recently, Watanabe et al. reported that the survival time of NHP recipients of lungs from GTKO/CD47/CD55 transgenic pigs was extended by 14 days (163, 180). These studies have demonstrated that the introduction of hCD47 can mitigate acute vascular rejection of lung xenografts and prolong porcine lung transplant survival time in NHP models. However, the limited survival time suggests the necessity of additional strategies in lung xenotransplantation.

Considering the above-mentioned achievements, heart and the kidney may be the first two solid organs to be used for clinical xenotransplantation. The modification of GTKO, hCRP, and hTBM may be indispensable in the donor pigs, at least for heart and kidney xenografts. In addition, a new problem of rapid and detrimental growth of xenografts after transplantation has emerged. This problem has been observed in both heart and kidney xenotransplantation (124, 161). The combination approaches of therapeutic reduction in blood pressure, reduction in corticosteroid dose, and administration of the mTOR inhibitor appeared to successfully prevent this problem. Moreover, the mechanisms of appropriate xenograft size require further study. The miniature pig as the donor animal may be necessary for the solid xenograft. Although some progress has been made in lung and liver xenotransplantation, survival time is limited, and preclinical results suggest that new genetic engineering and immunosuppression strategies must be developed before considering a transition to clinical trials.

## Porcine Endogenous Retroviruses in Xenotransplantation

A major concern in the field of xenotransplantation is the transmission of porcine pathogens to human recipients. Most porcine viruses, bacteria, and fungi can be eliminated by the selection of negative donor animals, breeding in sterile and isolated conditions, early weaning, and embryo transfer (181, 182). However, such strategies are impossible in the case of porcine endogenous retroviruses (PERVs) because PERVs are integrated into the porcine genome with multiple copies (183) and the number of PERV proviruses varies among pig breeds and organs, ranging from 1 to more than 100 (184). PERVs can be divided into three subtypes: PERV-A, PERV-B, and PERV-C (185). PERV-A and PERV-B are present in all pig breeds, whereas PERV-C is present in only some pigs (186). Recombinant virus PERV-A/C exhibits increased infectivity toward human cells (187). Therefore, the International Xenotransplantation Association recommends that the donor pig for xenotransplantation be free of PERV-C (188).

No consensus has yet been reached regarding whether it is necessary to guarantee PERV inactivation in donor pigs by genetic manipulation (189, 190). PERV transmission of pig-to-human and human-to-human cells was detected in several *in vitro* studies (108, 191). However, the infection was only observed in certain types of cells, as PERVs are unable to infect certain cell types because of the absence of a functional receptor on most cell surfaces (182). Furthermore, cellular restriction factors, such as APOBEC3G, play a key role in preventing PERV infection. Primary cells expressing APOBEC3G are difficult to infect. By contrast, HEK 293 cells, which are the most susceptible to PERV infection, do not express APOBEC3G (192).

To the best of our knowledge, PERV transmission has not yet been reported in preclinical pig-to-NHP models or in clinical cells transplantations to humans (193). If necessary, PERV inactivation can also be accomplished by genetically engineering pig donors. Early studies reported that PERV activation can be suppressed by RNA interference technology (194, 195). In 2015, Yang et al. inactivated all PERV-A and PERV-B genomes in PERV-C-free porcine cells using CRISPR/Cas9. The engineered cells reduced PERV transmission to human cells in *vitro* (196). In 2017, the same group inactivated all PERVs in a porcine primary cell line and generated healthy PERV-inactivated pigs through somatic cell nuclear transfer (108). These pigs provide a new strategy that eliminates the potential risk of PERVs in xenotransplantation. However, the susceptibility of these pigs to reinfection by PERV remains unclear. Godehardt et al. recently discovered that CRISPR/Cas9 PERV-inactivated cell line PK15 still produced impaired viral particles, although these virions were no longer infectious. The mutated PK15 cells are protected from the reinfection by PERV (197). However, these results were obtained through a monitoring period only up to 55 days, the reinfectivity remains a concern, and the persistent information and observation in PERV-inactivated pigs are necessary.

## Summary and Perspective

With recent achievements and the accumulation of experience with xenotransplantation in preclinical research, the first-in-human clinical trial may be possible in the near future. It is an inevitable trend that pigs modified with multiple genes are to be used as donor animals for xenotransplantation. New gene-editing technologies enable the production of multiple genetically engineered pigs in shorter periods of time and with greater efficiency. Various types of gene-modified pigs already exist, most of which are being tested in preclinical pig-to-NHP xenotransplantation models. In addition, new xenoreactive antigens are continually being discovered (198, 199), from which new knockout and transgenic pigs may be generated. Although assessment of current genetically modified pigs combined with immunosuppressive therapy in NHP models is complex and expensive, we agree with the opinion that substantial results should be obtain in NHP models before clinic application. Data and experience based on studies with NHP models suggest that combining various genetically modified pigs with different immunosuppressive therapies is necessary for the effective transplantation of different organs. Therefore, determining the optimal genetically engineered organ-source pig and immunosuppressive regimen strategy in pig-to-NHP models is a key step toward further clinical study.

Questions regarding the regulatory challenges and ethical concerns regarding clinical xenotransplantation are being asked worldwide. In 2003, the US Food and Drug Administration published comprehensive guidelines for xenotransplantation (http://www.fda.gov/cber/guidelines.htm). Scientists suggest that national regulatory authorities worldwide should reconsider guidelines and regulations regarding xenotransplantation so as to better enable design and safe conduction of informative clinical trials of xenotransplantation when supported by preclinical data (200). The current research makes some progress in meeting the criteria outlined by the recommendations of the International Society for Heart and Lung Transplantation published in 2000 (201). However, it is unclear which regulatory agencies consider current evidence to be sufficient for moving forward with clinical xenotransplantation.

An alternative potential approach that could alleviate the current shortage of human organs for transplantation is to create human–animal chimeras through various techniques, including stem cell biotechnology, regenerative medicine, and blastocyst complementation (202, 203). In addition, another approach to generating organs by 3D printing technology and decellularized scaffolds *in vitro* is currently available. Lee et al. described a 3D printing technique for building collagen scaffolds for the human heart spanning from the capillary scale to the full-organ scale. They demonstrated that cells could be embedded in the collagen to construct functional tissue and organs *in vitro* (204). To date, none of these approaches have reached the stage of testing on NHPs. We hope and believe that these approaches and xenotransplantation will complement each other in clinical application and collectively solve the problem of human organ shortage.

## Author Contributions

CQ and TL conceived the idea. TL wrote the manuscript. CQ, BY, and RW revised the manuscript.

### Conflict of Interest

The authors declare that the research was conducted in the absence of any commercial or financial relationships that could be construed as a potential conflict of interest.
